# The lab management practices of “Research Exemplars” that foster research rigor and regulatory compliance: A qualitative study of successful principal investigators

**DOI:** 10.1371/journal.pone.0214595

**Published:** 2019-04-24

**Authors:** Alison L. Antes, Ashley Kuykendall, James M. DuBois

**Affiliations:** Division of General Medical Sciences, Washington University School of Medicine, St. Louis, Missouri, United States of America; Mayo Clinic, UNITED STATES

## Abstract

**Introduction:**

Conducting rigorous scientific inquiry within the bounds of research regulation and acceptable practice requires a principal investigator to lead and manage research processes and personnel. This study explores the practices used by investigators nominated as exemplars of research excellence and integrity to produce rigorous, reproducible research and comply with research regulations.

**Methods:**

Using a qualitative research design, we interviewed 52 principal investigators working in the United States at top research universities and the National Institutes of Health Intramural Research Program. We solicited nominations of researchers meeting two criteria: (1) they are federally-funded researchers doing high-quality, high-impact research, and (2) have reputations for professionalism and integrity. Each investigator received an initial nomination addressing both criteria and at least one additional endorsement corroborating criteria 2. A panel of researchers and our research team reviewed the nominations to select finalists who were invited to participate. The cohort of “Research Exemplars” includes highly accomplished researchers in diverse scientific disciplines. The semi-structured interview questions asked them to describe the routine practices they employ to foster rigor and regulatory compliance. We used inductive thematic analysis to identify common practices.

**Results:**

The exemplars identified a core set of 8 practices and provided strategies for employing them. The practices included holding regular team meetings, encouraging shared ownership, providing supervision, ensuring adequate training, fostering positive attitudes about compliance, scrutinizing data and findings, and following standard operating procedures. Above all, the use of these practices aim to create a psychologically safe work environment in which lab members openly collaborate to scrutinize their work and share in accountability for rigorous, compliant research.

**Conclusions:**

Researchers typically receive limited systematic training in how to lead and manage their research teams. Training and education for principal investigators should include essential leadership and management practices and strategies that support doing high-quality research with integrity.

## Introduction

The United States invests billions of dollars in research annually [1–3]. An array of stakeholders have an interest in how this research is carried out [4]. Funding agencies and the public are primarily interested in advancing scientific knowledge and solving real-world problems. Institutions have an interest in attracting the highest caliber students and faculty to advance their missions, and a record of high achievement in research serves this end. Investigators are interested in research productivity, which allows them to secure additional resources to advance their research agendas. Junior researchers training with investigators want to learn the skills for success to advance their careers and research goals. Other researchers in the scientific community have an interest in the trustworthiness of research findings.

Addressing these stakeholder interests in the research enterprise requires attending to several dimensions of research ethics—or “doing good research in a good manner”—at the institutional level and within research labs and teams [4, 5]. In their labs, principal investigators (PIs) must foster research rigor and reproducibility, compliance with research rules and regulations, and effective workplace relationships. Increasingly, researchers are also called upon to ensure the social value of their research. These dimensions are important to justify the financial investment in research and the risks posed to animal and human subjects.

Long-standing scholarly study of science has presented key values and norms that guide the practice and ethics of research (e.g., accuracy, disinterestedness, honesty, trust, respect, and fairness) [6]. Other work suggests key characteristics of researchers (e.g., persistence and openness) [7, 8] and has elucidated situational influences (e.g., mentoring and competition) on scientific achievement [9]. It is in the day-to-day practice of science that individual characteristics, situational factors, and professional values operate to influence the behavior of researchers. However, recognition that the daily conduct of research often presents tensions between the ideals of science and actual practice [10, 11] has led, in part, to education in the responsible conduct of research as a key remedy to these issues [12]. However, reconciling these tensions and realizing optimal research practices also requires attention to the social and human dynamics within research labs.

Increasing attention to the interpersonal dynamics of effective research is reflected in the emergence of the “science of team science,” examining how research groups effectively perform research and foster interpersonal dynamics that facilitate good science [13, 14]. Concurrently, concerns about reproducibility and transparency in science [15] and failures to establish healthy research work environments [16–18] reflect additional attention to “doing good research in a good manner.” We contend that the decision-making and behavior of PIs, as the leaders of research teams, are at the heart of ensuring the best possible execution of science.

Thus, the purpose of this study was to identify the practices that “Research Exemplars”—PIs nominated by their colleagues as exemplary scientists and models of professionalism and integrity—employ to foster rigorous research and regulatory compliance within their teams. Our aim was to identify concrete approaches and strategies they employ to manage the responsible conduct of research in their groups. We apply a leadership and management framework to address our two primary research questions.

**RQ1:** What routine practices and habits do research exemplars utilize within their research teams to foster rigor and reproducibility?

**RQ2:** What routine practices and habits do research exemplars utilize within their research teams to foster research compliance?

Leadership and management are necessary when the efforts of people must be coordinated to achieve objectives [19]. When work is complex and innovative, and when it is high stakes, leadership is particularly critical to performance outcomes [20, 21]. Although these are central characteristics of the work of researchers, the role of PIs as leaders and managers in producing the highest quality research has received limited attention [22].

We define management and leadership in the following manner. Management focuses on *how* to do work; managers align processes and people so that work is consistent and efficient [23]. Leadership focuses on *what* to do and *why*; leaders articulate a vision and inspire people to work well together to do complex, innovative work [24]. In both roles, leaders and managers exercise influence; they influence people or work processes to achieve shared objectives [23]. An integrated model suggests the distinction between leadership and management roles is less important than achieving the right balance and enacting leadership behaviors and management structures that support efficiency and process reliability, human relations, innovation and adaptability [24].

The research setting may be one of the most complex domains in which to engage in leadership and management. PIs are often simultaneously required to control and monitor details to ensure accurate work, clarify rules to promote compliance with policies and standards, and emphasize hard work while striking a pace that balances speed and accuracy. They must also foster the engagement of staff and trainees, develop, motivate, and support personnel, and coordinate their team members’ shared efforts. Furthermore, PIs must innovate and anticipate long-term scientific agendas and compete successfully for funding [25]. Thus, a PI must engage in a variety of complex, multi-faceted behaviors, and know how to adapt to the tensions inherent in their roles—e.g., the need for control but also creativity; the need for detailed oversight of projects but also staff engagement and autonomy; the need for accurate but also quick work. Effectively executing these demands as a PI requires a deep behavioral repertoire and the ability to execute behaviors at the right time and in the right fashion [25].

The managerial and leadership behaviors identified in empirical research generally fit into two key domains. Task-oriented behaviors focus on providing structure and direction, for example clarifying roles, setting objectives, planning work, and coordinating tasks [24]. Relationship-oriented behaviors focus on ensuring the welfare and engagement of personnel, for example providing support, showing appreciation, providing mentoring, sharing in decision-making, and encouraging teamwork [24, 26]. Other leadership behaviors include those required for innovation, adaptability, and change, such as identifying new opportunities, building relationships with potential collaborators, and anticipating how a proposal will be evaluated by key constituencies [27].

It was our aim to identify the different practices and habits engaged in by PIs to foster high-quality research. We anticipated managerial, “task-oriented” behaviors to be particularly important in the domains of research rigor and compliance, as they require attention to detail, accuracy, consistency, and careful coordination of work activities [28–30].

## Methods

### Study design and participants

We employed an open-ended, qualitative methodology because we aimed to identify and relay concrete strategies employed by investigators to lead responsible research. We used purposive sampling to identify researchers representing a range of scientific disciplines who were exemplary in their scientific achievements and reputations for professionalism and integrity. Colleagues of the researchers provided initial nominations and secondary endorsements of their professionalism and integrity. A panel reviewed the nominations, and our research team reviewed their feedback and all of the nominations to select finalists. We conducted in-depth, semi-structured interviews with the participants we collectively refer to as “Research Exemplars.” The Washington University Institutional Review Board provided ethical approval for the study (IRB# 201601121).

### Nominations

We solicited nominations from the Carnegie-classified “highest” and “higher” research activity universities, accredited medical schools and schools of public health, and the National Institutes of Health (NIH) Intramural Research Program in the United States. We sent an initial email announcement and three reminder emails to nearly 1,500 academic deans, department chairs, and institutional research administrators (e.g., Vice Presidents for Research, Research Integrity Officers, Scientific Directors). We also emailed the PIs at institutions affiliated with the National Center for Advancing Translational Sciences’ Clinical and Translational Science Awards program [31]. The announcements invited the recipients to make a nomination and to forward the call for nominations to their colleagues.

The announcement indicated we sought nominations of federally-funded researchers in any career stage who lead a research lab or team. We indicated that the nominees must meet two criteria: (1) perform high-quality, high-impact research in any scientific discipline, and (2) have an outstanding reputation for professionalism and integrity. The email provided a web-link to additional information about the project and a nomination form. We indicated we would invite finalists to participate in a 1-hour interview about the practices they employ to lead their teams, and we would send them a plaque and post their biography on the project website (https://integrityprogram.org/exemplar-project/).

The nomination form asked the nominators to describe the nominee’s high-quality, high-impact federally-funded research program. Next, we asked for a specific description of how the nominee exemplified professionalism and integrity as a researcher. Finally, we asked the nominator to discuss how confident they were that their view was widely held by the researcher’s colleagues.

We also asked nominators to submit the nominee’s CV, and the contact information for three individuals who work closely with the researcher who we could contact for a secondary endorsement of the nominee. To obtain some verification that others held the same view of the nominee, we sent emails to the three individuals identified asking them to endorse the nominee if they agreed the researcher exemplified professionalism and integrity. The endorsers completed the two questions about how the researcher demonstrated professionalism and integrity and whether this view was widely held.

We asked the nominators and endorsers to characterize the nature and length of their relationship with the nominee. The nominators knew the nominees, on average, for 9.96 years (SD = 6.37) and 80% were institutional administrators, academic deans, or department chairpersons. Endorsers, on average, knew the nominees for 14.62 years (SD = 8.95) and 59% were faculty collaborators, and the remaining were department chairpersons, program directors, or trainees. We received 81 nominations, and 74 received at least one additional endorsement, qualifying them to move to the review panel. Before panel review, we emailed the nominees to confirm their interest in participating in the project if selected as finalists. All but one individual responded and agreed to participate, thus 73 nominations were sent to the panel for review.

### Panel review

The panel of volunteer reviewers consisted of 10 federally-funded researchers. We matched the reviewers’ scientific disciplines to the nominees’ fields, and two panelists reviewed each nomination. Five of the reviewers working in biomedical, public health, and social sciences reviewed the bulk of nominations (12 reviews each). The remaining three reviewers reviewed smaller sets of nominations from engineering, earth sciences, and physical sciences. Reviewers received the narrative information provided by the nominators and endorsers about the nominees’ research program and their professionalism and integrity. They also received the nominees’ CVs, and a summary of their academic rank, sources of funding, number of publications, and amount of grant funding (with a note that NIH intramural researchers do not seek external grants.)

We provided the review panelists with a one-page instruction sheet describing the two criteria for inclusion as an exemplar: scientific accomplishment as a federally-funded investigator and a reputation for professionalism and integrity. We instructed panelists that considerations for the expectation of high-quality, high-impact research might include: “receiving substantial grant funding, producing high-quality peer-reviewed journal articles, and addressing issues of social import.” Regarding the expectation of professionalism and integrity, we instructed panelists to consider “whether the characteristics or practices described in the materials are those that would be ideal for others in the scientific community to learn from or follow in their approach to lab management, mentorship, or leadership.”

Panelists scored the nominees on the two criteria using a 1 (*far below expectations*) to 7 (*far above expectations*) scale. We also asked reviewers to respond to the yes-no question, “Do you have any reservations about this person being identified as an exemplar?”, and to briefly comment on their ratings (and reservations, if necessary). We produced mean rating scores across the two criteria and two reviewers.

### Review by the research team

Our team (AA and JD) reviewed the panelists’ scores and comments, and we evaluated all of the nomination and endorsement narratives. We focused our review on nominees scoring at or above 5. The average reviewer rating of the final cohort of research exemplars was M = 6.02, SD = .60. Our aim was to verify, to the extent possible, evidence of behaviors or traits of the nominees that reflected professionalism and integrity. We examined the nomination and endorsement materials for examples of what set the researchers apart in how they approached doing their work.

It is of note that we did not define “professionalism and integrity” for nominators. Instead, we opted to allow nominators to make recommendations according to their views of professionalism and integrity in research. We found the exemplars were described as hardworking, enthusiastic, collaborative, meticulous, transparent, fair, and altruistic. Many held leadership positions at their institutions or in their fields. Nearly all were described as role models, advocates for others, or outstanding mentors. Additionally, exemplars were described as well-liked, highly-respected, and sought-after for their expertise and advice. The nomination and endorsement narratives offered examples of these qualities in practice; for example, taking the utmost care with their data and methods; being known for questioning their assumptions and bringing in outside perspectives; creating collaborative, engaged work environments; refusing to publish findings without confidence in their validity; being disciplined in requiring standardized procedures; discussing ethical considerations actively with their groups; sharing their data outside the lab; making sure team members receive due recognition; taking personal time to help colleagues and mentees.

In our assessment of the panelists’ feedback and the materials to select finalists, we aimed to ensure inclusion of researchers who met the criteria and who were diverse in research discipline (e.g., clinical, lab science, social/behavioral research), gender, and nation of birth. We sought at least 30 researchers, however, the high caliber of nominees ultimately led to 55 finalists. We emailed the 55 researchers selected as finalists, and all but three finalists agreed to enroll in the study and participated in the interview.

### Data collection

We conducted one-hour, semi-structured telephone interviews. Before completing the telephone interview, the participants received a consent document electronically and indicated consent to participate. Questions about the study were addressed via email or on the phone before interviews began. Participants also completed a brief online demographics survey and a short questionnaire about their work (e.g., hours worked per week and grant applications submitted per year).

The interview script consisted of 18 questions with follow-up prompts (S1 Appendix). First, the interview focused on rapport-building and background questions. Then, we told participants we wanted to understand the factors that contribute to the success and reputation for integrity of individuals nominated as research exemplars. We ask them to focus on things they do consciously to be the best researcher they can be, or things they do that others in their field might not do routinely. The next 11 questions asked about the individual traits, work strategies, and environmental or experiential factors that contributed to their success and reputation for integrity. The habits and routine practices questions focused on rigor and reproducibility, good team relationships, and compliance. Finally, we asked three questions about key career experiences and lessons.

We asked the participants to focus on work within their own research lab or team, even though they collaborate across labs and teams. We did not explicitly tell participants we were studying “leadership and management” to allow their leadership practices to emerge naturally. The interview script was developed by AA (an industrial-organizational psychologist with expertise in workplace psychology, leadership, and responsible conduct in research) [19, 32–34], and JD (a psychologist and bioethicist with expertise in research professionalism, research integrity, and professional decision-making, along with experience as PI of large federally-funded grants) [35–37]. Two public health graduate student research assistants conducted the interviews. They received 3 hours of training in interviewing practices and performed a practice interview observed by AA. After each interview, they documented how it went, and the team used this information to further standardize procedures between interviewers; although minimal problems arose. The interviews were digitally recorded and transcribed verbatim. They yielded 600 single-spaced pages of text.

### Data coding and analysis

We adopted a causation coding framework to identify causal explanations for why the exemplars had reputations for exemplary research [38], which we defined as fostering rigorous research, regulatory compliance, and good relationships [4]. In the interviews, we asked for the exemplars to discuss their own theory of what explains their success and reputations by asking them to identify the routine practices and habits they employed. In coding, we linked the practices they described to the outcomes of interest: rigor, compliance, and relationships. Our analysis of themes focused on building an understanding of the exemplars’, and our own, theory of the practices contributing to these outcomes. We anticipated leadership practices would emerge according to an existing broad framework of leadership behaviors: “initiating structure”—task-related behaviors focused on processes and procedures—and “consideration”—relationship-oriented behaviors focused on interpersonal aspects of leading people [26].

We developed codes to summarize concepts in the data inductively through several rounds of open-coding. First, AA and JD read the transcripts and marked emerging concepts to identify a coding framework consisting of parent codes (codes providing superordinate categories) for child codes (codes capturing specific concepts). The framework (S1 Table) included parent codes in several domains: e.g., research operations practices, relational and self-management practices, professional priorities, traits, and experiences. In addition, a parent-level “outcomes” code included the child codes of rigor and reproducibility, compliance, good relationships, balancing professional demands, and doing exemplary research. We linked practices to the outcomes when coding the excerpts of text containing practice codes by marking an accompanying outcome code each time we coded a practice. We only used the general “doing exemplary research” code when the practice discussed by the participant was not explicitly linked to the more specific outcome codes. We tracked outcome codes in this fashion to identify the casual links exemplars made between the practices and their intended outcomes.

After developing this initial codebook framework, one of the interviewers (AK) read 10 transcripts to identify the workability of the code framework and to elaborate on the child codes. The full team (AA, JD, and both interviewers) met to discuss and revise the codebook. Next, AK and AA tested the codebook on a second batch of 10 transcripts and continued to develop a comprehensive list of child codes. We also generated a codebook document with the definitions of the codes, example quotes illustrating each code, and rules for applying each code. This process repeated until we reviewed all transcripts and identified no more child codes. After each batch, team meetings focused on clarifying code definitions, rules for applying codes, and collapsing redundant codes.

We utilized Dedoose qualitative data analysis software [39] to train coders and perform coding. In Dedoose, coders mark excerpts of text within transcripts with codes representing concepts in those excerpts. This process generates present (1) and not-present (0) quantitative data for each of the codes within each transcript, and allows the textual data linked to the code to be downloaded for subsequent thematic analysis. To train on the final codebook and add rules to the codebook, AA and AK coded the same set of 10 transcripts and discussed. To obtain an estimate of reliability, they selected and both coded 50 chunks of narrative from transcripts across scientific disciplines, male and female participants, and across the 18 questions. Kappa, an estimate of inter-rater agreement, was .81. Kappa estimates of .61–.80 are considered very good, and above .81 excellent [40]. AA and AK then each proceeded to code 26 transcripts each. After coding 10 each, we selected a set of chunks, one from each of the 18 questions from across the transcripts, and recalculated Kappa (.73) to ensure we did not have major rater drift in applying the codebook.

The key purpose of this report is to identify practices linked to rigor and compliance. Thus, we examined each practice and their links with these outcomes by reading the excerpts for each practice to identify the themes represented within that concept. When practices linked to more than one outcome, we read and identified themes for each outcome. After reviewing the themes within excerpts for rigor and compliance, we reviewed the excerpts linked to the general “doing exemplary science” code to ensure that we had already identified all key ideas. During this process of analyzing themes associated with codes, we explored how the concepts and themes related to each other, identifying higher-level themes.

We report the frequency of all of the practice codes linked to rigor and compliance across the 52 exemplar transcripts, provide illustrative quotes, and elucidate the themes represented within the codes. We include specific strategies used by exemplars to provide practical insights. Our discussion focuses on the higher-order connections and key findings cutting across the results.

## Results

### Participant characteristics

We interviewed 52 researchers. The cohort performs diverse types of research, including basic lab research across a spectrum of life and physical sciences, clinical trials, behavioral interventions, engineering, informatics, and field research in disciplines ranging from earth science to virtual archeology. Thus, the cohort manages compliance in human subjects research, animal research, and laboratory safety. Table 1 describes the participants’ demographic and academic characteristics.

**Table 1:** Participant characteristics.

	No.	%
Gender		
Male	37	71
Female	15	29
Age		
30–39	2	4
40–49	12	23
50–59	20	39
60+	18	35
Born in the United States		
Yes	38	73
No^a^	14	27
Race		
White	45	87
Asian	4	8
Multiple	2	4
Not reported	1	2
Hispanic or Latino		
Yes	6	12
No	46	88
Highest Academic Degree^b^		
PhD only	37	71
MD only	9	17
MD and PhD	6	12
Academic Rank		
Associate Professor	5	10
Professor	43	83
Other (e.g., Senior Investigator; Lab Chief)	4	8
Type of Research^c^		
Wet Lab	26	51
Animal Subjects	25	49
Human subjects: clinical	21	41
Dry Lab	13	25
Human subjects: social or behavioral	12	24
Other (e.g., chemical synthesis, materials processing)	9	18
Discipline of Training		
Biomedical Sciences (biomedical, biological, and public health)	29	56
STEM Science (physical sciences, psychology, and earth sciences)	23	44

N = 52.

^a^13 (25%) reported English as their second language.

^b^84% earned in the United States.

^c^Participants selected “all that apply, thus % is > 100 (43% reported 1 type, 24% reported 2 types, and 33% reported 3 or more types).

Table 2 shows the exemplars’ career statistics and team characteristics. The researchers work with a mix of personnel on their teams, including high school students, undergraduate students, graduate students, post-doctoral researchers, research technicians, research coordinators, managers, and other full-time staff. Most (77%) work at public universities in the United States, and the remaining (15%) at private universities and NIH’s Intramural Research Program (8%). Of the exemplars at universities, about half (48%) were at medical schools and 73% were at “R1,” research highest activity, universities. The institutions represented the four geographic regions in the United States, the Midwest, Northeast, South, and West, as defined by the U.S. Census Bureau.

**Table 2:** Research exemplar career and team characteristics.

	M	SD
Exemplars		
Number of years doing research^a^	28.18	11.89
Number of research labs or groups worked in across career	6.45	4.83
Number of peer-reviewed publications	137.9	95.95
Amount of grant funding in career	$26,923,344	$26,155,063
Estimated hours worked per week	59.81	9.97
Research Teams^b^		
Number of personnel working in lab or research team	12.31	10.06
Number of projects running in lab/team at one time	7.23	4.72
Number of grant proposals submitted per year^c^	5.40	4.27
Number of papers submitted for publications per year	9.98	7.35
Days per week PI works in same space or building as research team	4.69	1.28

^a^defined as “years doing research that led to publications”.

^b^Participants were asked to characterize “on average”.

^c^N = 48, as NIH intramural researchers do not seek grants.

Our analysis of the interview narratives yielded 8 practices that 50% or more of the exemplars indicated foster rigor and/or compliance. It is of note that 4 of these same practices also linked to good relationships or balancing professional demands. We will describe the findings regarding relationships and professional demands in a companion report. We also found a second set of less frequently mentioned practices, including 8 practices focused on fostering rigor and 1 practice focused on compliance.

The practices offer ideals to strive for in leading a research team. However, it is important to note that not all exemplars engaged in all practices, and certainly, they did not execute the practices flawlessly. Many exemplars noted challenges they encounter, such as finding compliance burdensome, or struggling to identify what they should and should not delegate. The top practices are illustrated with quotes in Table 3.

**Table 3:** Lab management practices fostering rigor and compliance.

Practice	Linked Outcomes	Illustrative Quotes	No.	%
Hold regular team meetings	Rigor, Compliance, Relationships	“There’s nothing that’s hidden. We talk about data out loud. That lab meeting that we have each week is probably one of the most important hour to two hours that we spend together because everybody has to be able to show their raw data, everybody has to be able to talk about these difficulties.” (Participant 52)	43	83
		“It’s very important that the projects have regular meetings; they have clear agendas, and they have clear to do lists at the end of the meeting so that people are responsible and held accountable for going to the next step.” (Participant 21)		
Encourage shared ownership and decision-making	Compliance, Relationships, Balancing demands	“So there is less of a delegation, but more of a partnership…we need to do parallel things, and that’s the way to ensure rigorousness and integrity.… So, if I read a paper and miss something, another person is also reading the paper and will miss some other things. So, together we might be able to make up a whole picture.” (Participant 5)	38	73
		“With all of the human trial studies that we do, the students actually have to submit the IRBs and work with all of the clinicians that we’re working with…. So they’re in charge of it.… One of the biggest attributes we have…is actually giving…the sense of ownership, and once they have that sense of ownership and telling them these are the expectations, then they’re able to perform with good integrity.” (Participant 24)		
Provide supervision and guidance	Rigor, Compliance, Relationships	“Listening to the people who are in the laboratory and doing things. So if they’re running into something that’s a barrier for them, listening to what that barrier is, and number one, seeing if there’s a solution to get around that barrier, or number two, telling them why it’s there so that we have a check point.” (Participant 17)	37	71
		“I do try to talk to all of my people every day. I think the more junior people…even if it’s just ‘good morning, do you have a good plan for what you’re going to do today?’ Even if that’s all it is, they know that, number one, I care…that I’m available for questions, comments, concerns. They know that what they’re doing is being supervised.” (Participant 41)		
Ensure sufficient training	Rigor, Compliance	“We show someone, teach them, they do it while we watch, then they do it themselves, and then they do it themselves again and we come back and check later. So part of it is in the way we train people.” (Participant 37)	35	67
		“She [the lab manager] handles getting people to the person who’s going to train them.… Once they’re trained, they get a document from the trainer saying they know how to handle that piece of equipment and training completed is entered on the spreadsheet. So, that’s just to get started. The first few times that you do a procedure, especially in animals, like the first 5 times in animals, someone is watching…and checking off a checklist.” (Participant 34).		
Foster positive attitudes about compliance	Compliance	“I think personal relationships, not to treat the IRB as a thing but as people…getting to know them, inviting them to our lab, when we have a difficult decision, to really have them involved with us where we get their feedback.… They understand our work, and we’re open about where we have a fork in the road and getting their feedback, I think that makes a real difference.” (Participant 21).	34	65
		“A lot of people will have, attack the messengers. I think it’s much better to be working with them, and working out protocols together and always being open and transparent about what we plan on doing.” (Participant 23)		
Scrutinize data and findings	Rigor	“I do meet with people basically weekly to look at data, and to go through data…I try very hard…if the data doesn’t show the result that I expected or wanted, to try not to react to it and personally attack the person…frequency of interacting with people around primary data is important, and being open to hear results, whatever they are, even if they’re not what you expected or wanted…” (Participant 11)	32	61
		“You really have to have access to the raw data and you have to sit down and talk about the data interpretation with the students and make sure that you understand how they’ve reached their conclusions, how they’ve interpreted their data, any additional data that they’ve collected that they’re not showing you that might change the interpretation.” (Participant 20).		
Express values and expectations	Rigor, Compliance, Relationships	“I also let my laboratory know…it doesn’t matter how long it takes us to do something, we’re gonna do it right, because we’re going to be able to say to people we have confidence in what we do, because here’s what we’ve done to get there.” (Participant 52)	31	60
		“…we’re strict in terms of getting the work done and that the work has to be high quality, but…I like to see the people in my lab working with a smile on their face, preferably enjoying what they do, and not feeling like I’m a task master…” (Participant 16)		
Establish and follow SOPs	Rigor, Compliance	“…there are standard operating procedures that everybody has to be familiar with, and then…making sure that not only have they read it the first time, but…go over it again…” (Participant 7)	26	50
		“We use SOP’s for everything, absolutely everything, and people come to our lab and they’re just blown away by the fact that we have, we have a map to show you where to park.… So that just makes it easier.” (Participant 37)		

### Hold regular team meetings

Discussed by 83% of exemplars, the cornerstone of rigorous research is holding regular meetings. As one exemplar put it: “…the first round of vetting of what they have accomplished is through our internal group meeting or lab meeting” (Participant 41). Nearly all exemplars hold weekly meetings. Meetings serve several distinct purposes.

Meetings, first and foremost, provide the opportunity to share data. This fosters transparency about data and permits team members to scrutinize results. This routine helps lab members feel comfortable putting their data, findings, and interpretations on display for others to critique. It creates a norm of openness about data, helps to identify potential problems in the data, and fosters a sense of team collaboration on projects. Several exemplars emphasized that a PI must never make lab members feel that they will be unhappy with the data presented to them. One noted, “…I let them know that progress isn’t getting the results I want…progress is getting the results that the experiment gives us” (Participant 17).

A related purpose of meetings is to provide updates, coordinate, and identify potential problems with a study that impact the quality of data or compliance with a scientific or ethical protocol. Meetings allow team members, particularly when several must coordinate to execute protocols, to double-check procedures and talk about concerns. These meetings also allow investigators to understand what has been accomplished and allow the team to plan. The exemplars talked about using these meetings to make sure they are fully aware of how the research is going and to create accountability for next steps. Several PIs mentioned the importance of agendas, to-do lists, and action steps to foster awareness and accountability among team members. For example, one exemplar described requesting an outline of goals and progress: “They have to prepare a write up every single time that we meet…essentially outlines their goals, and then the next time, what they met on those goals” (Participant 51).

Whether it relates to ensuring sound, high-quality research, or compliance with regulations and ethical standards, the exemplars repeatedly emphasized that people must be comfortable communicating about mistakes. Overall, communication at team meetings must be transparent and open. Exemplars stressed that people must feel the environment is “non-threatening.” A few exemplars even described sharing their own mistakes and trials to model openness. One exemplar put it this way:

I tell [my team] that the only mistake that can’t be fixed is the one they don’t tell me about…if they’ve done something wrong, or they’ve discovered that something went wrong…I want to know about it.… I don’t get angry…because everyone’s human and we all make mistakes, including me. The only thing I ever get angry about is if I discover that they’ve hidden something from me. So, transparency and displaying that transparency is really key.…

(Participant 4)

Exemplars also hold meetings to discuss new study designs and plan out study protocols that have not yet launched. Occasionally, meetings serve the purpose of developing or training lab members by discussing new topics or having outside experts come to present on new regulations or important compliance issues.

### Encourage shared ownership and decision-making

Another top practice, shared by 73% of exemplars, was establishing an environment where everyone shares in responsibility for compliance and integrity. For example, one strategy was to design compliance protocols together as team. This not only ensures that everyone knows what each team member is responsible for, but also that the protocol can be discussed and revised if a problem arises.

Another aspect of shared ownership was appointing a compliance point person. Many exemplars described someone they rely on as a source of expertise and consistency on matters of compliance. Typically, this person was an experienced member of the lab or designated compliance manager. The point person helps the PI oversee compliance matters and double-check procedures and documentation. A point person might perform specific checks of protocol compliance, for example, they might regularly review documentation like protocol checklists and signed informed consents. Moreover, this team member serves as the go-to person for daily compliance questions or issues. One exemplar described it this way: “…different staff or the research faculty are in charge of various components of the laboratory…they serve as an additional, immediate onsite resource for laboratory processes in a way that I can’t, given my administrative duties” (Participant 10).

Other than selecting an experienced individual, some PIs mentioned appointing a person who is good with details, or a “perfectionist.” This person then contacts the PI when needed. Also, the point person is often involved in training newcomers and ensuring they are knowledgeable of the rules. One exemplar relied on a graduate student in this role: “…I also have a safety officer in the lab…it’s an advanced grad student…and he’ll basically train any new student coming in on a one-on-one basis…go through all of the things that we need to check” (Participant 45).

### Provide supervision and guidance

The next practice of utmost importance mentioned by the majority (71%) of exemplars was providing comprehensive, ongoing supervision and feedback to their students and personnel. Meetings provide an oversight mechanism for PIs, but exemplars also emphasized oversight and feedback more generally. They described regular, typically daily, interactions with smaller groups and individual team members. They make sure that they are interacting with and communicating with everyone on their team. One described it simply this way: “…I go to the lab every day and engage all of them” (Participant 38).

At its core, this practice is about being an involved and engaged PI and mentor. The exemplars value awareness of how the research is going day-to-day so they can provide timely guidance and direction. The form that oversight took varied by level of the student or staff member: daily more intensive supervision for newcomers, inexperienced staff, or a struggling trainee; regular “check-in” meetings with more experienced individuals; and one-on-one meetings with the most senior personnel on an as-needed basis. Above all, their objective was to interact with everyone to identify potential problems and help identify ways to overcome them. One exemplar noted: “I’m meeting with them…and making sure there aren’t problems, that if there is a problem, they’re bringing it to my attention” (Participant 43).

Regarding rigor specifically, exemplars described being involved in and overseeing the execution of research and the production of data. Through their awareness of how data collection is progressing, they can help trouble-shoot when studies are not going according to plan, or when they or lab members identify inconsistencies in data. For those doing experimental work, this typically meant they were in and out of the lab interacting with people. Those doing clinical work emphasized being sure that people know they are available. Further, they are regularly in contact with lab members, so they can learn immediately if there are any questions, concerns, or needs for guidance about protocols. Access to the PI was of critical importance; typically, PIs interacted daily with their teams. Some exemplars mentioned that they have an “open door” or share their calendar with their team. Other PIs noted it is imperative that their team works in a space in close proximity to their office.

Similar to their oversight of research and data collection, engagement as a supervisor allows their teams to address problems related to compliance as quickly as possible. Often, supervision related to compliance focused on checking in with the compliance point person and establishing the procedure that dictates how the compliance person communicates concerns directly to the PI. Several exemplars expressed the importance of being mindful about the possibility that compliance or research integrity issues could arise, like compliance protocol violations, plagiarism, or data manipulation, and of being vigilant in monitoring for them. They described not being suspicious, but mindful.

Several exemplars specifically noted the importance of supervision and feedback for new members to the team and for people who are doing new, unfamiliar tasks. Through regular interaction, the PI gains confidence that lab members are prepared to work more independently. The PIs noted that regular supervision and feedback are the key means to develop trust in the knowledge and skills of their team members, which ultimately allows them to delegate and share in decision-making and ownership.

It is worth noting that the supervisory role of a PI evolves. With a smaller lab, or at an earlier career stage, some described being extremely involved, even working alongside students in the lab. With time, a PI’s involvement in the smallest details may diminish, but this heightens the need for regular interaction, feedback and open lines of communication. Also, PIs with large teams or labs may oversee managers or coordinators who help to provide some of the supervisory support to others. However, the exemplars described being both highly engaged in communicating with those managers or coordinators while also still interacting regularly with all the people on their teams.

Overall, the investigators described being aware, being present and available, and interacting regularly with individuals. Approachability, in particular, was a prominent theme in their responses. Most exemplars explicitly noted that people should feel any concern or question may be brought to their attention at any time. They consistently reiterated the importance of openness and communication. By knowing the day-to-day activities of the team, they can provide timely, effective guidance and help their teams overcome challenges.

Importantly, some exemplars explicitly pushed back against the idea that this kind of engagement and regular contact with people is “micromanagement.” Many felt that through their engagement on matters of data quality and compliance, they created the sense that every team members should play a role in monitoring and creating checks and balances about data and compliance. Furthermore, many noted that problems, mistakes, and concerns should be approached in an evenhanded fashion. One stated: “I think the key is to make sure that I am talking to them, and I’m not judging them or evaluating them when we talk. It’s all about discussion…” (Participant 39). Another noted: “If you yell at people, if you brand them as incapable researchers…then people will go into safe mode. If they go into safe mode, they are not really a scientist anymore…” (Participant 6). Finally, another exemplar reflected on the aim of establishing openness: “I want to create an environment of openness with my students so that they’re able to present to me the truth and not be afraid that I’m going to get angry” (Participant 35).

### Ensure sufficient training

Another common practice noted by 67% of exemplars was the need to ensure adequate training of newcomers to their research teams to foster rigor and compliance. Several mentioned the adult learning heuristic ‘tell, show, do’—tell someone how to do a task, then show them how to do it, and then watch them as they practice doing it. Some described assigning tasks with specific metrics or outcomes they could check to ensure the person knew how to carry out the task accurately. Others described using a tiered, experience-based system in which experienced lab members train the beginners while the mid-level trainees observe. Later, mid-level trainees may become the experienced trainers.

Several exemplars noted the need to draw on the help of lab members to train others, and to formalize training as much as possible, especially as the lab gets bigger and the PI accumulates greater responsibilities. Several exemplars noted using training guides and standardized training manuals to ensure that everyone is taught to do things the proper, rigorous way. Regarding compliance, several noted that they do not assume that people know the rules, and they make sure that team members meet the institutional compliance training requirements. Other strategies for training on compliance-related matters were to work together on regulatory applications, have occasional special meetings about regulatory issues, or to bring in outside regulatory experts if extra training needs arise. One exemplar relayed their approach as follows:

We have a monthly staff meeting, and we talk about compliance and regulations. I will send reminders as needed about the importance of compliance with regulations, compliance with our research protocols with the university requirements and regulations, and we discuss a lot of that also when we’re writing proposals.

(Participant 12)

Another described building staff knowledge through experience with compliance protocols:

I require all of them to write and submit IRB or IACUC application, of course with my supervision, so that we all will be familiar with the issues, not just in terms of research, but also in treating humans and animals in ethical ways. I also require them to personally get trained in every aspect of research regardless of whether they’re going to do that type of work in their research or not.

(Participant 38)

Overall, the PIs described the critical need to ensure that no one, whether an undergraduate, graduate, or post-doctoral researcher, performs a task until they reach adequate mastery. Without this level of mastery, the team risks wasting resources, producing bad data, or failing to ensure compliance. By attending carefully to training, the PI develops trust in their team members, allowing them to work independently. Notably, several investigators mentioned that, because the period of necessary learning before being productive is extensive, they ask people to commit at least two years in their lab.

### Foster positive attitudes about compliance

In line with the idea that shared ownership fosters compliance, 65% of the exemplars described the need to foster positive attitudes about regulatory compliance. This included several elements. First and foremost was the need to have good relationships with regulatory agencies and institutional officials. PIs noted the importance of viewing these officials as helpful experts rather than antagonizers. As noted above, some even reported inviting such experts into their labs to give presentations or share best practices. They noted the value of viewing compliance experts as part of a team’s competitive advantage, that is, viewing them as a resource to support the good work of the team, rather than criticize it. However, this particular strategy will be most effective at institutions with compliance offices that have robust educational and support services.

Another element of this practice was to provide continued professional development around issues of compliance; for example, by dedicating a lab meeting to discussing changes in regulations or to discussing a case example. Keeping the importance of compliance salient in the lab, particularly by making compliance a topic of regular discussion, allows the team to integrate compliance into how they naturally go about their work. An exemplar described encouraging awareness as follows: “We just have kind of ‘safety moments’ and safety updates every couple of months to make sure that everybody is remembering the proper set of procedures to go through if there’s an emergency in the lab…” (Participant 11).

Additional strategies for fostering a culture of compliance were to set expectations of compliance by explicitly insisting that lab members follow the rules, and to make compliance easy, or the default approach to doing science. For example, this could be achieved by using standard operating procedures (SOPs) and ensuring adequate training. Several exemplars went even further, noting that the team must value doing what is ethically best for patients, animals, lab, or science, even when it is above and beyond the requirements. Finally, even when the PI finds compliance matters a burden, several exemplars noted it is important not to communicate that attitude to team members.

### Scrutinize data and findings

The exemplars described meticulousness and skepticism towards raw data and their team’s interpretations of findings. This strategy was mentioned by 61% of exemplars. They sought to cross-check data and findings as thoroughly as possible and to ensure a culture of transparency about data. To achieve this, they urged people to talk about data regularly. They encouraged lab members to show each other their data to create a culture in which people are comfortable talking about data. Exemplars ensure that lab members participate in both receiving and offering feedback so that they become comfortable with critique. One PI noted people should never be afraid to show their data: “I tell them I want all data. There is no good data, there is no bad data unless somebody makes a decision…I am ultimately responsible, or…the team is.… You have to have all original data.” (Participant 6).

Some exemplars described bringing in outside colleagues who can offer fresh perspectives on their findings or inviting others to collaborate, especially those who they know will disagree, in order to generate a greater level of scrutiny. Their aim of setting a tone of objectivity and scrutiny towards data was especially achieved through regular meetings and interactions about data. Many exemplars described that this mindset carries beyond meetings to the way members of their teams approach data as individuals, and in the way they work together collaboratively outside of meetings to ensure the best research.

### Express values and expectations

We heard, from 60% of exemplars, about the significance of a PI explicitly stating what is important as the team goes about their research. That is, what the PI values and, in turn, what the PI expects. To foster rigor, the exemplars communicate high standards. Specifically, they express the importance of doing research accurately, meticulously, transparently, and with integrity. They expect lab members to work hard, but they also tell them not to sacrifice quality by cutting corners or rushing. Several exemplars mentioned the need to maintain the credibility of the lab, noting they must always be able to defend their results with confidence. Furthermore, PIs tell lab members it is essential to maintain openness and objectivity as researchers. One exemplar described the mindset they expect from team members in the following way:

I try to, on a daily basis, remind my students…be very, very skeptical…it starts first with each individual being skeptical of their own data, and not assuming anything. Never assume anything. That’s the fatal flaw in a scientist is to assume that you know what you’re doing.

(Participant 22)

Included in this notion of objectivity was the idea that lab members must never feel that the PI expects or demands a particular result. Furthermore, several exemplars noted the need to explicitly tell lab members that they must be willing and able to admit mistakes, and it is ok to “be human.”

Similarly, PIs express high standards regarding compliance. We heard from several exemplars that they tell lab members that the rules are important, and that PIs insist members make it a priority to know and follow the rules. In addition, exemplars state the consequences of compliance failures: that is, they tell team members that the privacy of participants, or the safety of fellow lab members, is at stake. Importantly, many noted the need to state these expectations particularly when a new member starts in the lab, and not to assume that people know the rules and expectations. Some exemplars also explicitly expected everyone to work together as a team to ensure all lab members understand and follow the rules. For example, some exemplars appointed existing lab members to help newcomers learn the rules. Other strategies to reinforce the importance of compliance were to have compliance concerns as the first agenda item at meetings and to ask new members to sign an agreement stating their commitment to following the rules.

### Establish and follow standard operating procedures

Another practice, mentioned by 50% of the exemplars, was to establish and follow standard operating procedures (SOPs) for scientific and compliance-related procedures. Exemplars stated it is unwise to take for granted that everyone understands how to perform procedures, or that they reliably remember every step. As one put it:

…we write protocol manuals…we actually list out every step of what someone is supposed to do as they go through the study…so everything is laid out. We don’t assume that people know or will always remember every single step. So there’s a checklist for every protocol that we use, and people have to sign off.

(Participant 4)

Exemplars described the importance of highly specific SOPs and the need to use them for everything in their research. SOPs provide PIs with some peace of mind that lab members will consistently perform procedures. They also ensure that best practices in the lab will not be lost over time as members of the lab change. Moreover, SOPs provide a means to discuss and update scientific protocols and regulatory compliance in meetings, particularly to troubleshoot problems. Some exemplars noted drawing on the guidance and expertise of compliance offices to develop or update SOPs.

As illustrated in Table 4, the remaining set of 9 less frequently mentioned practices emphasized procedures to ensure their teams carry out research in a meticulous and coordinated manner. This set of practices focused exclusively on rigor, except for one practice related to compliance.

**Table 4:** Lab management practices fostering rigor and compliance.

Practice	Linked Outcomes	Illustrative Quotes	No.	%
Verify findings	Rigor	“Well, when you do an experiment you get a result, and you interpret it, and you think you know what it means.… But often your initial inclination about what the result means is not correct when you’ve done more experiments or thought about it more…that all goes to not being afraid of disproving your hypothesis because if you’re afraid to disprove it then you get a nice result, and what you want to do is make some fancy conclusions and publish your results in a fancy journal, and the motivation is not to try to disprove it. If you’re a rigorous scientist then you need to do what’s necessary to try to disprove it.” (Participant 18)	25	48
		“I have typically another set of hands to try to reproduce what someone else has done. I need to have at least two sets of hands being able to do this and we are constantly talking about reproducibility and how one does that themselves and with someone else.” (Participant 52)		
Document procedures	Rigor	“We work at the level of good laboratory practice, which is a lot of record keeping. Making sure everything is calibrated properly and everything is measured accurately, and done with study plans, study reports, in a blinded manner, in a very exacting manner.” (Participant 34)	23	44
		“I try to instill in all of my students to have a very high level of detail in putting down important information that later becomes an experimental procedure directly from their notebook.” (Participant 45)		
Design scientifically sound studies	Rigor	“Being very rigorous in how you develop a project and how you go about defining the experimental variables that you’re going to measure, doing appropriate control to make sure that what you think you’re seeing is as accurately supported as possible, to include appropriate statistical guidelines, to make sure you’re reproducing the findings with enough independent observations, to use as many objective criteria as possible when we are developing, forming, and analyzing the results from our experiments.” (Participant 46)	21	40
		“We spend a lot of time talking about control experiments…the control experiments are maybe not always the most exciting experiments to do, but they’re usually the most important experiments to do, because that’s where you really begin to see what’s unique about the results that you’re putting forward.” (Participant 20)		
Coordinate the study team	Rigor	“We put the [study plan] table…up on the screen and we all look at it together, because everyone on that team will be doing a part of the research…it has to be really well orchestrated.… The devil’s in the details…many little things you can work out if the whole team is sitting together and they each have input about how they’re going to do their portion of it.” (Participant 34)	17	33
		“Whenever we are planning an experiment we all sit down as a group and go through it and say here’s what we’re asking, here’s what we need to do, and we sort of say who’s going to do what.” (Participant 2)		
Handle data properly	Rigor	“All of the files, they’re all kept on Dropbox where everybody has access. We have a folder where all of our code is and anybody is supposed to be able to go in and grab the data and be able to run the same sort of analysis.” (Participant 8)	16	31
		“We, of course, do regular backups and we then do backups of the backups.” (Participant 6)		
Involve multiple researchers on projects	Rigor	“…you would like to have more than one person do a particular key study…basically having collaborations within your laboratory, and that’s really one of the strong sides of my lab, that my people work with each other in many, many different angles…they see then what the results are and there’s a feedback…” (Participant 13)	13	25
		“Having multiple team members on the same project ensures the quality of the analysis, thereby allows identification of any gaps in the scientific account.” (Participant 27)		
Share data outside the lab	Rigor	“how do you ensure integrity?…I would say presenting…bring in even the opposition, try and co-op them, but during the process itself, you should present your preliminary works at international meetings and serendipitous things can happen, people…make important criticisms or positive contributions…put the work out there to your peers from around the world.” (Participant 14)	11	21
		“…we’re in the process of getting all of those data up there [in a data repository], available, documented so that other people can use them…so anybody else who wants to use it, if they really want to reproduce anything they have to be able to access it…get the data out that they need…so that other people can use and combine what they have with what we have, and use our code and that kind of thing to really advance science.” (Participant 8)		
Report findings completely and accurately	Rigor	“…I want a perfect, you know, description of the experimental data…it needs to be full, fully characterized…so I try as hard as I can to hold people…responsible for, you know, for getting completeness of data.” (Participant 15)	9	17
		“Being very careful in the methodology and very clear about what methods are used.” (Participant 28)		
Advance compliance through audits	Compliance	“…we’ll do a routine check…a couple of times a year… just because we’re not perfect, they’ll reveal that there was an issue with X or there was an issue with Y…we report it to our IRB of course, that there might have been a violation to specific study procedure or a certain form wasn’t collected how we said it was going to be collected in IRB protocol, and then we identified this because of our routine checks, and then we figure out, okay, this happened, and…now how can we prevent this from happening again?” (Participant 44)	9	17
		“There are safety inspections and that’s a part of what we do…so people are aware of that so that’s an extra motivation to make sure there’s good compliance.” (18)		

### Verify findings

We heard of the need to verify findings from 48% of exemplars. For those doing experimental work, in particular, it was essential to verify findings through repeated experiments, and often through having multiple people replicate experiments. As one expressed: “…you repeat it, and you repeat it…we want to be sure…repeat is the mother of success” (Participant 13). Exemplars recommended finding help outside the lab if the lab is having trouble replicating. They also shared the importance of digging into results when they find something they expected, not only when they find something they did not expect. They stated the lab must figure out whether their result is real or not. One exemplar described it as follows:

…being extremely slow and methodical and careful and going back and checking and rechecking and looking at things from different perspectives, bringing in a lot of people to consult, others to independently replicate your results, others to still independently replicate those. I’ve never put anything out that hasn’t been replicated at least twice independently by people.

(Participant 14)

The bottom line was to be skeptical of what they think they have in front of them and to look at their findings from new angles, for example, to repeat experiments, assess new controls, or look to other studies to triangulate their findings.

Verify findings was mentioned by about half of exemplars, and most investigators meant replicating findings. It might be expected that replicating would be mentioned more frequently, but we think this may be a function of two factors. First, the investigators were conducting diverse types of research: some were doing basic laboratory studies where explicit replication is customary, whereas others performed clinical or behavioral research and explicit replication of datasets is not possible. Instead, in these fields “scrutinizing data and findings” captures the standard approach to validating findings. Second, we asked investigators to describe practices they thought others in their field might not do routinely, which may have led them to focus less on practices they potentially viewed as typical.

### Document procedures

We heard from 44% of exemplars that rigorous research requires meticulous, constant documentation of every decision made, even the smallest detail. One investigator noted: “I also ask my students, even though we’re a computational group, I ask them to maintain a research log, a lab notebook” (Participant 9). Whether documentation takes the form of lab notebooks, written plans, detailed notes, or metadata, exemplars conveyed that everything should be documented and researchers should plan ahead for documentation in their studies. There should be an expectation that personnel keep detailed records and notes about virtually everything. A few PIs provided the advice to give students or staff example lab notebooks, research reports, or lab meeting agendas and meeting minutes to illustrate the desired format and detail.

### Design scientifically sound studies

Another issue raised by 40% of exemplars was that rigor starts at step one. They described the need to spend the necessary time to plan everything as carefully as possible from the outset. As one PI put it: “You think before the study, and you don’t think during the study, or you think only if you have to, as needs arise” (Participant 42).

Ensuring that their team planned the most scientifically sound, rigorous designs included several elements. A solid scientific foundation was one concern. They noted the need to study the literature to determine a theoretical framework, conceptual model, or what findings are already available. This allows the team to make sound research design decisions and find creative ways to extend and improve upon what already has been done. A few PIs noted using outside guidelines and checklists for rigor available from agencies like the NIH and journals. Some also described going outside their teams to obtain external feedback and criticism on ideas and study methods from the very beginning. Finally, specific study design considerations included the following: blinding, the use of controls, validating measures and techniques, calibrating equipment, having multiple methods of data collection to triangulate findings, and having repeated measures of the same data point. Several described thinking backwards; beginning with the end goal in mind and then thinking backwards to design studies. Above all, they reiterated that during the study design phase, PIs must not be afraid of findings that run counter to their hypotheses.

### Coordinate the study team

Most of the exemplars have multiple team members working together to carry out studies. As alluded to in other practices, the need to coordinate the activities of the team is essential, and thus was explicitly noted by 33% of exemplars. This practice emphasized assigning and discussing roles down to the minute details from the very beginning of the process. This practice included getting the team together at the beginning of the project to map out goals and roles. As noted by one exemplar: “…we are planning an experiment, we all sit down as a group and go through it and say here’s what we’re asking, here’s what we need to do…who’s going to do what…” (Participant 5).

Then, it was essential to repeat check-ins during data collection and when creating a data analysis plan, executing analysis, or drafting results. Coordination and specification of roles was particularly important in how some of the exemplars ran their large research teams because they had students doing research while being supervised by post-doctoral researchers. Thus, this coordination and delineation of roles was important so that people knew who to contact for immediate, day-to-day guidance when doing their research.

### Handle data properly

Finally, 31% of exemplars emphasized the importance of recording, storing, and backing up data appropriately. The PIs described storing data in a central place (often a shared drive) that is accessible to them at all times (and often to multiple lab members) and over a long period of time. They insisted that all data be stored in this central location, or that there be documented justification for anything that is not archived or is missing. They felt multiple researchers should be involved to improve data integrity—that is, typically, just one individual should not regulate data collection, analysis, and storage. Some PIs had co-leads on projects, and they made practices like double-data entry and group data analysis the norm. One investigator described data management this way:

…I always tell my students…back up all your data…at least in two places…and everyone is able to go into every single user’s directory…we are all collaborative, we look at everyone’s raw data in the analysis…and we maintain the data as long as we can.

(Participant 9)

### Involve multiple researchers on projects

Many practices implied the importance of teamwork, but several investigators (25%) explicitly noted the importance of internal collaboration for rigor and integrity. By having multiple people work on the same or parallel projects, access to, and review of data, are not isolated to one person, improving the integrity of the project. Strategies included “co-leads” of projects, group data entry and analysis, and the explicit expectation of co-authorship in their group. This approach lends to a collaborative environment and better research because cross-checks and feedback are built into the scientific process in the lab. One even noted the practice can impact the experiments researchers take on: “They also help each other so they co-author on each other’s papers, and so they’re a little less afraid…to do a labor extensive experiment because they know that they can have help for it” (Participant 35). It was noted, however, that this approach may be less feasible in smaller groups.

### Share data outside the lab

Some exemplars (21%) discussed sharing their data or findings outside their lab early and throughout the research process. Exemplars felt this transparency promotes accountability, reproducibility, rigor, and, overall, advances science. Critique from other investigators on emerging findings could be obtained by presenting at conferences, or by seeking out the input of other investigators. Sometimes this latter strategy includes inviting outside collaborators to join their projects. One exemplar described this practice as follows:

…show the data to others and get feedback from people who may be naïve so that we can get a fresh and unbiased perspective, because if you’re only talking to people in your group you can get a bit myopic…it’s essential to take your findings outside of the group, ideally when they’re still in their preliminary stages so you can get feedback as the work is developing.…

(Participant 46)

Sharing also included submitting data or methods to repositories, as one exemplar described:

…you have to get your data onto some kind of data repository and have it well explained enough that other people can understand what it is and make use of it…that even goes to code…everything you do is potentially retrievable and can be checked. It adds a burden to us, but I think it’s really a good thing, and it really kind of increases the value of the work if you can document everything and put everything out there…

(Participant 16)

### Report findings completely and accurately

Another practice shared by 17% of the exemplars was to ensure as much certainty in findings as possible before publication. This included, at times, explicitly ensuring they did not rush to publication. As one noted, “…we are careful not to talk about our results until we have confidence in them…I tend to be conservative in not publishing too quickly…we’re telling a story in each paper, and is the story really clear?” (Participant 21). They also discussed that it is essential to report all findings completely and precisely.

### Advance compliance through audits

Finally, 17% of the exemplars mentioned using compliance audits to their advantage as an opportunity to increase awareness in their group and improve practices. Some referred to required audits, and specifically included everyone in the lab. One exemplar stated: “…we’ve had audits and I’ve had everybody be part of the audit because we don’t have anything to hide, although obviously there may be some questions” (Participants 4). Others used internal audits conducted on a regular basis by their team to identify areas for improvement in their processes.

## Discussion

We found the research exemplars create an open, transparent environment to foster the highest quality research. Their research teams openly discuss problems, scrutinize their data, methods, and findings, and work cooperatively to do sound science and ensure research compliance. The need for transparency and openness to foster reproducibility in the broader scientific community has received a great deal of attention [15, 41]. Our findings identify several concrete strategies used by highly successful researchers for fostering openness and transparency as they lead their teams. It is important to note that not all exemplars mentioned every practice and specific applications of the practices may vary by context (e.g., type of research or number of personnel). However, several critical practices clearly emerged.

At the core of creating and maintaining an exemplary environment was regular, open communication in team meetings, along with frequent interaction between PIs and team members. These dyadic interactions include the PI actively offering guidance and feedback as an engaged mentor and supervisor, as well as team members actively approaching the PI. This latter form of interaction requires that team members find the PI approachable and accessible. The exemplars were both physically present in their labs, and they allowed people to feel psychologically safe to disclose mistakes and problems.

Psychological safety exists in a work group when people feel they can speak up and share ideas, ask questions, state concerns, or disclose mistakes without fear of penalization or people thinking less of them [42, 43]. Mutual trust and respect characterize psychologically safe environments, and psychological safety is linked with workplace engagement, creativity, high performance, learning, safety, and ethical behavior [44–46]. Exemplars emphasized the need to be open to findings that do not support their hypotheses, and the need to respond evenhandedly to mistakes. More broadly, exemplars fostered a collaborative spirit among their team members.

Another important practice was encouraging shared ownership and decision-making in their teams. This “collective” approach to leadership draws on the skills and expertise of team members to ensure effectiveness, rather than concentrating authority exclusively with one individual [47, 48]. For example, some exemplars relied on compliance “officers” in their groups to help ensure adherence to rules and procedures. More broadly, they set a positive tone about compliance and encouraged everyone to recognize their personal responsibility for compliance. We also found that exemplars placed great emphasis on scrutinizing data and findings. By explicitly communicating their values and expectations, exemplars could intentionally establish a workgroup environment in which people share assumptions about responsible and desired approaches to going about the work [21, 49].

Not surprisingly, ensuring that research personnel have the skills to execute tasks competently was among the top practices. We heard of the importance of being intentional and systematic about training, ensuring an individual has demonstrated competence and reliability. The implementation of SOPs was another strategy for managing effectiveness and consistency of high-stakes tasks. A collective model of leadership requires drawing on the unique skills and expertise within the group, but of course, this does not obviate the accountability of a central leader to ensure effective functioning of the group [48]. Thus, PIs must identify the talents of others, develop skills where needed, and empower properly trained team members to take on increasing responsibility.

The final set of practices relayed by exemplars complements the foregoing practices. Exemplars described the need to ensure excellent study design, verification of findings, meticulous documentation, proper data management, sharing of data, and precise reporting of findings. This set of recommendations reiterates a key principle for responsible research and data management, namely meticulousness in all stages of the research, from study design and planning to data collection, storage, and sharing [50, 51]. While the precise implementation of such practices varies by type of research, the key “quality assurance” principle urges investigators to produce, process, and report on data within a set of defined, controlled conditions [52–54]. We anticipate increasing focus on data sharing practices, wherein investigators are called on to implement practices that enhance the discoverability and usability of their data for the broader scientific community [55].

We anticipated management behaviors—those task-oriented practices that structure the operations of a research lab and provide personnel with direction and clarity in expectations—to be especially important for rigor and compliance. Indeed, many of the practices shared by exemplars are “classic” management activities—facilitating meetings, providing supervision, training personnel, structuring and standardizing procedures. However, interpersonal relationships and communication were at the core of most of these activities. We learned about “partnership,” “harmony,” “respect,” and “trust” among team members being central to high-quality research. Exemplars carry out their management activities in a way that engenders engagement and cooperative teamwork. They were mindful of the critical importance of interpersonal dynamics and positive team relationships in their labs and how these interactions support good science [56]. We also learned of the intentionality behind the broader culture that was developed within their teams.

Overall, our findings align well with contemporary models of leadership that assert effective leadership may not reside within any one person, but it is a function of effective information exchange, shared decision-making, and problem-solving in a collaborative manner that capitalizes on the expertise and skills with the team [48]. However, for maximal effectiveness, a PI must build the right team, develop talent within the team, clarify the mission, establish positive team interactions, and ensure opportunities for open communication [21, 48].

Our findings are also consistent with key recommendations for responsible mentoring which emphasize that a mentor should serve as a role model of high standards and ensure proper training in research practices and research integrity [5, 57]. Specifically, these recommendations state that mentors must communicate the importance of responsible research practices, meet regularly with trainees and personnel, review data and work for accuracy, and provide SOPs for research operations [50, 57]. Effective mentor-mentee relationships require mutual trust and respect, and mentors are expected to offer support and encouragement [57]. It has also been noted that explicitly articulating the values underlying practices in the research group is important for conveying and clarifying the standards for responsible research and reduces misunderstandings about expected behaviors [58]. However, while mentoring is an important lens through which to view the role and responsibilities of an effective PI, it is an incomplete one. A broader understanding of the responsibilities of PIs must take into account their role in establishing a thriving work environment and facilitating effective collaboration among team members.

### Implications for training

Establishing management systems and structures requires time and intentionality on the part of PIs when they establish their labs. Early-career researchers need guidance in this regard [19, 59]. This guidance may be especially important as they likely feel pressure for immediate research productivity relative to taking time to establish workgroup structures. It is inefficient for every researcher to start up a lab and recreate organizational structures and processes, often learning solely by trial and error [19]. A set of best practices for lab start-ups would facilitate establishment of sound procedures. Practical information, such as templates for developing protocols, lab manuals, or meeting agendas, could be provided to investigators to encourage them to adopt practices that facilitate research excellence, or simply to streamline their startup.

Investigator leadership training should also provide guidance in how leadership behaviors form a workplace culture, along with how to establish good dynamics among members of a team. Our findings suggest that researchers need outstanding communication and interpersonal skills. The team-oriented nature of contemporary research [13] and the importance of high-quality interactions for responsible research suggest the need for researchers to learn about these human dynamics alongside scientific methods. Again, we expect practical guidance to be particularly valuable; for example, brief, structured guides for starting conversations about values and expectations or about integrity in research. It may also be necessary to provide strategies for navigating personal challenges and pressures, such as tools for time and stress management. The ability to successfully navigate professional challenges, make prudent decisions, and effectively lead others, rely, at least in part, on the ability to manage personal pressures [36].

We also think that approaching such training programs in a comprehensive manner—framing them as fostering excellence in research—will be most attractive to investigators. Specifically, programs should communicate that rigorous, high-quality research, regulatory compliance, ethical research, mentoring behaviors, and interpersonal dynamics are intersecting domains with cross-cutting leadership skills and responsibilities. For example, fostering respectful workplace relationships and a positive team culture are central to all these ends. Additionally, having the right set of management structures and the right culture allows a leader to free up time and energy to focus on other highly valued tasks, like grant writing and strategic planning.

At present, education in the responsible conduct of research (RCR) serves as a key method to encourage researchers in the training stages of their careers to adopt responsible practices and values that support ethics in research. Importantly, not all junior faculty researchers are required to receive RCR education at that career stage (i.e., not all are “NIH-funded trainees”, for instance, on a career development award). However, training is needed when making the transition from doing research inside another investigator’s group to leading one’s own research team. Rather than describing this training as “RCR education,” it may be more effective to refer to it as “leadership development.” There are some existing programs in mentoring skills and lab management [59, 60], but such programs generally remain optional.

Within existing RCR education that tends to reach graduate students and post-doctoral researchers, trainees should learn about the role of the work group environment and interpersonal dynamics in responsible research. Research trainees may not be able to overtly influence the research environments in which they work, but they may be able to influence them in subtle ways. Furthermore, all trainees could be encouraged to actively consider the practices they are exposed to and whether they might be ideal, or less than ideal, to adopt in the future. Finally, all trainees could benefit from learning interpersonal and intrapersonal skills that support effective workplace interactions [56].

### Limitations

Our findings should be examined in light of potential limitations. Institutional culture may play a role in leadership practices that are viewed as effective and yield success; our findings do not speak to this issue. However, we drew investigators from a diverse set of universities and fields to increase the generalizability of our findings, but our findings do not address the role of institutional culture or differences by types of research fields. In fact, institutional culture may have influenced those who were nominated; for example, we did not receive any nominations from Ivy League institutions. Additionally, we report on the practices the exemplars described employing. However, it is possible that some of their responses reflected their intentions more than their actual practices. Yet, their remarks still reflect those practices they view as ideals.

Additionally, we attempted to verify to the extent possible that the participants met the inclusion criteria of professionalism and integrity. However, this was not possible to establish with complete certainty. Our approach included receiving at least two recommendations for each individual, and the nominators and endorsers knew the researchers for many years. It is also of note that many exemplars freely remarked about their own shortcomings and challenges; thus, it appears they were introspective and open with us. However, in future work, it may be ideal to also speak with individuals working inside the research teams of investigators to gain their perspectives. Research examining their perceptions of psychological safety, for example, would be valuable.

Additionally, our call for nominations indicated that we would consider nominees in any career stage, but the investigators included in our final cohort were senior (i.e., associate or full faculty rank). This is a strength in that we obtained the views of highly experienced individuals but a limitation in that they had already experienced the challenges and pressures faced by junior faculty. Future work might focus on understanding the specific challenges and pressures faced by individuals earlier in their careers. Relatedly, the well-established investigators likely had greater resources and staffing relative to those earlier in their careers; this may influence the leadership dynamics in their groups. Nonetheless, we think that the practices reported by exemplars can be applied in any research group. Training programs could help investigators understand nuances in adopting practices with different types of staffing or resources.

Overall, we think that the practices of this cohort of exemplars are noteworthy because of their diversity in types of research and institutions, and the rigorous process we used to obtain the sample. We sought an outstanding group for our interviews, those whom colleagues viewed as exemplary on two criteria: excellence in science and integrity and professionalism. Our process required each individual to receive a nomination and endorsement, and the nominees were reviewed by a panel of researchers. The high reviews given by the panel suggest our process generated the high-caliber group we sought. After talking with them, we found strong patterns in their responses. Moreover, we had a sizable sample for a qualitative interview study. Often samples of 30 [61] or even 12 [62] are viewed as sufficient to identify common themes in a study of this kind. Moreover, many practices identified in this study are those absent in the labs of researchers referred for lapses in research compliance and integrity [63], suggesting their importance.

## Conclusion

We identified practices, along with specific strategies, employed by successful PIs for leading their research labs. The scientific community would benefit from greater awareness of the role of leadership and management in performing research. We recommend additional research and the development of evidence-based educational interventions to meet the needs of investigators.

## Supporting information

S1 Appendix Interview guide. The full semi-structured interview guide (lightly edited for length). The themes reported in this manuscript arose primarily in response to questions 7 and 9. However, rigor and compliance-related responses emerged throughout the interview and were coded accordingly.
(DOCX)

S1 Table Coding scheme. The full project coding scheme demonstrating the parent code categories and the specific child codes (coders also had definitions, examples, and rules to guide application of the codes). This report describes the codes under *Research Operations Practices* and *Relational and Self-Management Practices* that linked to the outcome codes *Rigor* and *Compliance*.
(DOCX)

## References

[1] National Institutes of Health. Budget 2018 [cited 2018 July 12]. https://www.nih.gov/about-nih/what-we-do/budget#note.

[2] Centers for Disease Control and Prevention. FY 2017 budget and appropriations snapshot. 2018 Contract No.: CS283043-B.

[3] President signs omnibus appropriations bill into law; Federal agencies funded through Sept. 30 [Internet]. 2017; May 8, 2017. https://www.nsf.gov/about/congress/115/highlights/cu17_0508.jsp

[4] DuBois JM, Antes AL. Five dimensions of research ethics: A stakeholder framework for creating a climate of research integrity. Acad Med. 2017;93(4):550–5. Epub 2017/10/27. 10.1097/ACM.0000000000001966 29068823 PMC5916747

[5] National Academies of Science. On being a scientist: A guide to responsible conduct in research. Washington DC: National Academics Press; 2009. 25009901

[6] Allchin DJS, Education. Values in science: An educational perspective. 1999;8(1):1–12.

[7] Hermanowicz JC. What Does It Take to Be Successful? Science, Technology & Human Values. 2006;31(2):135–52. 10.1177/0162243905283637

[8] Feist GJ. A meta-analysis of personality in scientific and artistic creativity. Personality and social psychology review. 1998;2(4):290–309. 10.1207/s15327957pspr0204_5 15647135

[9] Mumford MD, Connelly MS, Scott G, Espejo J, Sohl LM, Hunter ST, et al Career Experiences and Scientific Performance: A Study of Social, Physical, Life, and Health Sciences. Creativity Research Journal. 2005;17(2–3):105–29.

[10] Anderson MS, Martinson BC, De Vries R. Normative dissonance in science: Results from a national survey of U.S. scientists. Journal of Empirical Research on Human Research Ethics. 2007;2(4):3–14. 10.1525/jer.2007.2.4.3 19385804

[11] De Vries R, Anderson MS, Martinson BC. Normal misbehavior: Scientists talk about the ethics of research. Journal of Empirical Research on Human Research Ethics. 2006;1(1):43–50. 10.1525/jer.2006.1.1.43 16810336 PMC1483899

[12] Kalichman MW. Responding to challenges in educating for responsible conduct of research. Acad Med. 2007;82(9):870–5. 10.1097/ACM.0b013e31812f77fe 17726394

[13] Bennett LM, Gadlin H, Marchand C. Collaboration and team science: A field guide. 2nd ed: NIH National Cancer Institute, Center for Research Strategy; 2018 139 p.

[14] National Research Council. Enhancing the Effectiveness of Team Science. Washington, DC: The National Academies Press; 2015. 26247083

[15] Munafò M, Nosek BA, Bishop DVM, Button KS, Chambers CD, Percie du Sert N, et al A manifesto for reproducible science. Nature Human Behaviour. 2017;1 10.1038/s41562-016-0021. PMC7610724 33954258

[16] Norris D, Dirnagl U, Zigmond MJ, Thompson-Peer K, Chow TT. Health tips for research groups. NATURE. 2018;557:302–4. 10.1038/d41586-018-05146-5 29769687

[17] Van Noorden R. Some hard numbers on science’s leadership problems. NATURE. 2018;557:294–6. 10.1038/d41586-018-05143-8 29769686

[18] Else H. Does science have a bullying problem? Nature. 2018;563:616–8. 10.1038/d41586-018-07532-5 30487619

[19] Antes AL, Mart A, DuBois JM. Are Leadership and Management Essential for Good Research? An Interview Study of Genetic Researchers. Journal of Empirical Research on Human Research Ethics. 2016;11(5):408–23. 10.1177/1556264616668775 . 27646401 PMC5182150

[20] Vessey WB, Barrett JD, Mumford MD, Johnson G, Litwiller B. Leadership of highly creative people in highly creative fields: A historiometric study of scientific leaders. The Leadership Quarterly. 2014;25(4):672–91.

[21] Robledo IC, Peterson DR, Mumford MD. Leadership of scientists and engineers: A three‐vector model. Journal of Organizational Behavior. 2012;33(1):140–7.

[22] Mumford MD, Peterson D, Robledo I. Leading Scientists and Engineers: Cognition in a Socio-Technical Context In: Hemlin S, Allwood CM, Martin B, Mumford MD, editors. Creativity and Leadership in Science, Technology, and Innovation. London: Routledge; 2013 p. 29–57.

[23] Yukl G. Leadership in Organizations. 8th ed Boston MA: Pearson; 2013.

[24] Yukl G, Lepsinger R. Why integrating the leading and managing roles is essential for organizational effectiveness. Organizational Dynamics. 2005;34(4):361–75. 10.1016/j.orgdyn.2005.08.004

[25] Lawrence KA, Lenk P, Quinn RE. Behavioral complexity in leadership: The psychometric properties of a new instrument to measure behavioral repertoire. The Leadership Quarterly. 2009;20(2):87–102. 10.1016/j.leaqua.2009.01.014.

[26] Lambert LS, Tepper BJ, Carr JC, Holt DT, Barelka AJ. Forgotten but not gone: an examination of fit between leader consideration and initiating structure needed and received. The Journal of applied psychology. 2012;97(5):913 10.1037/a0028970 22708919

[27] Antes AL, Schuelke MJ. Leveraging technology to develop creative leadership capacity. Advances in Developing Human Resources. 2011;13(3):318–65. 10.1177/1523422311424710

[28] Begley CG, Buchan AM, Dirnagl U. Robust research: Institutions must do their part for reproducibility. Nature. 2015;525:25–7. 10.1038/525025a 26333454

[29] Sonstein SA, Seltzer J, Li R, Silva H, Jones CT, Daemen E. Moving from compliance to competency: a harmonized core competency framework for the clinical research professional. Clinical research. 2014;28(3):17–23.

[30] Jefcoat AM. The ‘Triad of Noncompliance’ as a tool for understanding, preventing and correcting protocol and procedural noncompliance. Lab animal. 2008;37(10):459–63. Epub 2008/10/01. 10.1038/laban1008-459 . 18810264

[31] Institute of Medicine. The CTSA program at NIH: Opportunities for advancing clinical and translational research. Washington, D.C.: The National Academies Press; 2013. 24199260

[32] Antes AL, Brown RP, Murphy ST, Waples EP, Mumford MD, Connelly S, et al Personality and ethical decision-making in research: The role of perceptions of self and others. Journal of Empirical Research on Human Research Ethics. 2007;2(4):15–34. Epub 2007/12/01. 10.1525/jer.2007.2.4.15 . 19385805

[33] Antes AL, Chibnall JT, Baldwin KA, Tait RC, Vander Wal JS, DuBois JM. Making professional decisions in research: Measurement and key predictors. Accountability in research. 2016;23(5):288–308. 10.1080/08989621.2016.1171149 . 27093003 PMC4968873

[34] Antes AL, Mumford MD. Strategies for leader cognition: Viewing the glass "half full" and "half empty". The Leadership Quarterly. 2012;23(3):425–42. 10.1016/j.leaqua.2011.10.001

[35] DuBois JM, Chibnall JT, Tait RC, Vander Wal JS. The Professionalism and Integrity in Research Program: Description and preliminary outcomes. Acad Med. 2017 Epub 2017/06/20. 10.1097/acm.0000000000001804 28640035 PMC5738297

[36] DuBois JM, Chibnall JT, Tait RC, Vander Wal JS, Baldwin KA, Antes AL, et al Professional Decision-Making in Research (PDR): The validity of a new measure. Science and Engineering Ethics. 2015;22(2):391–416. 10.1007/s11948-015-9667-8 26071940 PMC4819725

[37] DuBois JM. Is compliance a professional virtue of researchers? Reflections on promoting the responsible conduct of research. Ethics & behavior. 2004;14(4):383–95. Epub 2010/01/08. 10.1207/s15327019eb1404_8 . 16625734

[38] Saldaña J. The Coding Manual for Qualitative Researchers. 3 ed Seaman J, editor. Thousand Oaks, CA: Sage Publications Ltd; 2016.

[39] Dedoose. Version 8.0.35 web application for managing, analyzing, and presenting qualitative and mixed method research data,. 2018;Los Angeles, CA: SocioCultural Research Consultants, LLC, www.dedoose.com.

[40] Landis JR, Koch GG. The measurement of observer agreement for categorical data. biometrics. 1977:159–74. 843571

[41] Nosek BA, Alter G, Banks GC, Borsboom D, Bowman SD, Breckler SJ, et al Scientific standards. Promoting an open research culture. Science. 2015;348(6242):1422–5. 10.1126/science.aab2374 . 26113702 PMC4550299

[42] Carmeli A, Reiter-Palmon R, Ziv E. Inclusive leadership and employee involvement in creative tasks in the workplace: The mediating role of psychological safety. Creativity Research Journal. 2010;22(3):250–60. 10.1080/10400419.2010.504654

[43] van der Rijt J, van de Wiel MWJ, Van den Bossche P, Segers MSR, Gijselaers WH. Contextual antecedents of informal feedback in the workplace. Human Resource Development Quarterly. 2012;23(2):233–57.

[44] Baer M, Frese M. Innovation is not enough: Climates for initiative and psychological safety, process innovations, and firm performance. Journal of Organizational Behavior. 2003;24:45–68. 10.1002/job.179

[45] Carmeli A, Sheaffer Z, Binyamin G, Reiter-Palmon R, Shimoni T. Transformational leadership and creative problem-solving: The mediating role of psychological safety and reflexivity. The Journal of Creative Behavior. 2013;48(2):115–35. 10.1002/jocb.43

[46] Edmondson AC, Lei Z. Psychological safety: The history, renaissance, and future of an interpersonal construct. Annual Review of Organizational Psychology and Organizational Behavior. 2014;1:23–43. 10.1146/annurev-orgpsych-031413-091305

[47] D’Innocenzo L, Mathieu JE, Kukenberger MR. A Meta-Analysis of Different Forms of Shared Leadership–Team Performance Relations. J Manage. 2014 10.1177/0149206314525205

[48] Friedrich TL, Vessey WB, Schuelke MJ, Ruark GA, Mumford MD. A framework for understanding collective leadership: The selective utilization of leader and team expertise within networks. The Leadership Quarterly. 2009;20(6):933–58.

[49] Harris SG. Organizational Culture and Individual Sensemaking: A Schema-based Perspective. Organ Sci. 1994;5(3):309–21.

[50] Steneck NH. ORI introduction to the responsible conduct of research. Updated ed. Washington, D.C.: U.S. Government Printing Office; 2007 xiii, 164 p. p.

[51] Schreier AA, Wilson K, Resnik D. Academic research record-keeping: Best practices for individuals, group leaders, and institutions. Acad Med. 2006;81(1):42–7. 16377817 10.1097/00001888-200601000-00010 PMC3943904

[52] Chavan GBJS. Implementation of Good Laboratory Practices (GLP) in basic scientific resaerch: Translating the concept beyond regulatory compliance. Regulatory Toxicology and Pharmacology. 2017;89:20–5. 10.1016/j.yrtph.2017.07.010 28713068

[53] International Conference on Harmonisation. Good Clinical Practice (ICH GCP) 1.34 Investigator Geneva: International Conference on Harmonisation; 2016 [cited 2016 December 8]. http://ichgcp.net/4-investigator.

[54] Baker M. How quality control could save your science. Nature. 2016;529:456–8. 10.1038/529456a 26819028

[55] Wilkinson MD. The FAIR Guiding Principles for scientific data management and stewardship. Scientific Data. 2016;3. 10.1038/sdata.2016.18 PMC4792175 26978244

[56] Antes AL, DuBois JM. Cultivating the Human Dimension in Research. Molecular Cell. 2018;72(2):207–10. 10.1016/j.molcel.2018.09.015. 30340021 PMC6529938

[57] Shamoo AE, Resnik DB. Responsible conduct of research. 3rd ed New York: Oxford University Press; 2015.

[58] Bird SJ. Mentors, advisors and supervisors: Their role in teaching responsible research conduct. Science and Engineering Ethics. 2001. 10.1007/s11948-001-0002-1 11697001

[59] Seeliger J. Scientists must be taught to manage. Nature. 2012;483(7391):511 10.1038/483511a 22460862

[60] Mentoring matters. Nature Cell Biology. 2010;12(2):101-. 10.1038/ncb0210-101 20118995

[61] Corbin JM, Strauss AL. Basics of qualitative research: Techniques and procedures for developing grounded theory. 4th ed: Sage publications; 2015 456 p.

[62] Guest G, Bunce A, Johnson L. How many interviews are enough? An experiment with data saturation and variability. Field methods. 2006;18(1):59–82.

[63] DuBois JM, Chibnall JT, Tait RC, Vander Wal JS. Lessons from researcher rehab. Nature. 2016;534:173–5. 10.1038/534173a 27279195

