# Supplementary material for: The lab management practices of “Research Exemplars” that foster research rigor and regulatory compliance: A qualitative study of successful principal investigators
Source: PLoS One. 2019 Apr 24;14(4):e0214595. doi: 10.1371/journal.pone.0214595 (PMC6481787; doi:10.1371/journal.pone.0214595)
Supplement: S1 Appendix — The full semi-structured interview guide (lightly edited for length). The themes reported in this manuscript arose primarily in response to questions 7 and 9. However, rigor and compliance-related responses emerged throughout the interview and were coded accordingly. (DOCX) [file pone.0214595.s001.docx]

| **Questions** | **Probes** |
| --- | --- |
| **Part A.** Introductory statement, purpose of study, and verbal consent for recording | |
| **Part B.** Background information and rapport-building | |
| 1. What motivated you to pursue a career in research? | *None* |
| 1. How would you describe the research that you do to a lay audience? | *None* |
| 1. What is the nature of your research team or lab? | Do you have staff, students, etc. who you work with and how many? |
| 1. What are key ethical, compliance, and/or social issues that arise in your research? | *None* |
| **Part C.** Factors contributing to the success and integrity of research exemplars | |
| 1. How would you describe your research personality? What key traits do you have as a researcher*?* | Do any other characteristics come to mind? |
| 1. How do you think these traits have contributed to your success as a researcher and reputation for integrity? | Can you speak to both success as a researcher and reputation for integrity? |
| 1. What are some of the habits or routine practices you have developed to foster scientific rigor and reproducibility in your lab? | Can you tell me more about that? How does that foster scientific rigor and reproducibility*?*  Do any other habits or practices come to mind? |
| 1. What are some of the habits or routine practices you have developed to support good working relationships in your lab? | Can you tell me more about that? How does that support good working relationships?  Do any other habits or practices come to mind? |
| 1. What are some of the habits or routine practices you have developed to ensure compliance, for example, compliance with regulations, ethical rules, or good clinical practice in your lab? | Can you tell me more about that? How does that ensure compliance?  Do any other habits or practices come to mind? |
| 1. What social responsibilities do you think you have as a researcher, and how do you engage these issues? | Are there other ways you engage these issues?  Do any other responsibilities come to mind? |
| 1. How do you manage your multiple obligations and workload? When you are juggling multiple priorities, how do you make decisions about what to focus on? | Can you describe other practices that you have found are essential to managing your time? |
| 1. How do you manage stress or pressure in your work? | *None* |
| 1. How do you find the right balance between detailed management and delegation to ensure efficiency and productivity while also ensuring quality and integrity? | *None* |
| 1. When you encounter uncertainty about what to do regarding a particular situation or problem in your professional work, how do you address this? | *None* |
| 1. How does your institution support your work? | *None* |
| **Part D.** Key experiences, factors, and lessons from exemplar’s career | |
| 1. What key events, situations, or experiences have shaped your approach to how you conduct yourself as a professional? | *None* |
| 1. Are there any other factors that have contributed your success that we have not yet discussed? | Is there anything else you want to tell us before we ask our final question? |
| 1. What is the top lesson or recommendation you would communicate to a new investigator about to embark on a career in research? | Is there another lesson you would like to share? |
