# Supplementary material for: The lab management practices of “Research Exemplars” that foster research rigor and regulatory compliance: A qualitative study of successful principal investigators
Source: PLoS One. 2019 Apr 24;14(4):e0214595. doi: 10.1371/journal.pone.0214595 (PMC6481787; doi:10.1371/journal.pone.0214595)
Supplement: S1 Table — The full project coding scheme demonstrating the parent code categories and the specific child codes (coders also had definitions, examples, and rules to guide application of the codes). This report describes the codes under Research Operations Practices and Relational and Self-Management Practices that linked to the outcome codes Rigor and Compliance. (DOCX) [file pone.0214595.s002.docx]

| **Parent code: RESEARCH OPERATIONS PRACTICES** |
| --- |
| **Child codes** |
| Advance compliance through audits |
| Clarify research focus |
| Coordinate the study team |
| Design scientifically sound studies |
| Document procedures |
| Ensure sufficient training |
| Establish and follow SOPs |
| Handle data properly |
| Hold regular team meetings |
| Involve multiple researchers on projects |
| Report findings completely and accurately |
| Scrutinize data and findings |
| Share data outside the lab |
| Verify findings |
| **Parent code: RELATIONAL AND SELF-MANAGEMENT PRACTICES** |
| **Child Codes** |
| Address interpersonal conflict |
| Adopt prioritization and planning techniques |
| Cultivate a positive team environment |
| Encourage shared ownership and decision-making |
| Engage in self-care |
| Express values and expectations |
| Foster positive attitudes about compliance |
| Hire team members cautiously |
| Lead by example |
| Manage emotions and reframe thinking |
| Provide supervision and guidance |
| Recognize cross-cultural dynamics |
| Seek advice |
| Tailor approach to individual needs |
| **Parent code: OUTCOMES** (Link practices to one or more of the outcomes child codes) |
| **Child codes** |
| Rigor and reproducibility |
| Compliance |
| Good team relationships |
| Balancing professional demands |
| Doing exemplary research |
| **Parent code: ETHICAL, LEGAL, OR SOCIAL ISSUES ENCOUNTERED** |
| **Child codes** |
| Advocate for science |
| Animal Care |
| Community engagement |
| Conflicts of Interest |
| Data integrity and reproducibility |
| Environmental protections |
| Helping people/doing socially important work |
| Honest reporting of findings |
| Human cells research |
| Human subjects research ethics |
| Interpersonal relationships |
| Mentor next generation of scientists |
| Occupational safety |
| Participate in policy development |
| Plagiarism |
| Promote diversity in science |
| Steward of public funds |
| **Parent code: PROFESSIONAL PRIORITIES** |
| **Child codes** |
| Conducting collaborative research |
| Discovery and challenge |
| Engaging communities and doing socially important research |
| Ensuring a reputation for quality work |
| Fostering good relationships |
| Helping people and having a positive impact on society |
| Mentoring and developing personnel |
| Planning for long-term sustainability and success |
| Promoting diversity in science |
| **Parent code: OTHER PROFESSIONAL ROLES/RESPONSIBILITIES** |
| **Child codes** |
| Administrative |
| Clinical responsibilities |
| Mentoring |
| Peer review |
| Teaching |
| **Parent code: BARRIERS/CHALLENGES** |
| **Child codes** |
| Compliance burden |
| Lack of funding/resources |
| Lack of training |
| Other job responsibilities |
| Personal shortcomings |
| **Parent code: TRAITS** |
| **Child codes** |
| Balancing/reconciling opposites |
| Careful/cautious |
| Caring/compassionate |
| Creative/innovative |
| Curious |
| Detail-oriented |
| Discerning |
| Easygoing |
| Flexible |
| Focused |
| Hardworking |
| Honest |
| Humble |
| Impatient/Urgency |
| Objective/impartial/open-minded |
| Passionate |
| Persevering |
| Praising/Positive |
| Resilient |
| Responsible/dependable |
| Self-confident |
| Self-reflective |
| Workaholic |
| Works well under pressure |
| **Parent code: EXPERIENCES** |
| **Child codes** |
| Bad colleague/mentor |
| Educational experience |
| Engaged in self-learning/no formal training |
| First or early success |
| Great colleague/collaborator |
| Great mentor or role model |
| Post-doc or career development experience |
| Previous job/field |
| Traveled abroad |
| **Parent code: ENVIRONMENT** |
| **Child codes** |
| Current institution |
| Family of origin/upbringing |
| Nation of birth |
| Supportive family/spouse |
| **Parent code: APHORISMS AND ADVICE** |
| **Child codes** |
| Aphorisms – their own |
| Aphorisms – attributed to others |
| Advice |
